# Molecular Modeling and Functional Analysis of Exome Sequencing–Derived Variants of Unknown Significance Identify a Novel, Constitutively Active FGFR2 Mutant in Cholangiocarcinoma

**DOI:** 10.1200/PO.17.00018

**Published:** 2017-08-01

**Authors:** Jan B. Egan, David L. Marks, Tara L. Hogenson, Anne M. Vrabel, Ashley N. Sigafoos, Ezequiel J. Tolosa, Ryan M. Carr, Stephanie L. Safgren, Elisa Enriquez Hesles, Luciana L. Almada, Paola A. Romecin-Duran, Eriko Iguchi, Aryan Ala’Aldeen, Jean-Pierre A. Kocher, Gavin R. Oliver, Naresh Prodduturi, David W. Mead, Asif Hossain, Norine E. Huneke, Colleen M. Tagtow, Sikander Ailawadhi, Stephen M. Ansell, Michaela S. Banck, Alan H. Bryce, Estrella M. Carballido, Asher A. Chanan-Khan, Kelly K. Curtis, Ernesto Resnik, Chelsea D. Gawryletz, Ronald S. Go, Thorvardur R. Halfdanarson, Thai H. Ho, Richard W. Joseph, Prashant Kapoor, Aaron S. Mansfield, Nathalie Meurice, Amulya A. Nageswara Rao, Grzegorz S. Nowakowski, Animesh Pardanani, Sameer A. Parikh, John C. Cheville, Andrew L. Feldman, Ramesh K. Ramanathan, Steven I. Robinson, Raoul Tibes, Heidi D. Finnes, Jennifer B. McCormick, Robert R. McWilliams, Aminah Jatoi, Mrinal M. Patnaik, Alvin C. Silva, Eric D. Wieben, Tammy M. McAllister, Kandelaria M. Rumilla, Sarah E. Kerr, Konstantinos N. Lazaridis, Gianrico Farrugia, A. Keith Stewart, Karl J. Clark, Eileen J. Kennedy, Eric W. Klee, Mitesh J. Borad, Martin E. Fernandez-Zapico

**Affiliations:** **David L. Marks**, **Tara L. Hogenson**, **Anne M. Vrabel**, **Ashley N. Sigafoos**, **Ezequiel J. Tolosa**, **Ryan M. Carr**, **Stephanie L. Safgren**, **Elisa Enriquez Hesles**, **Luciana L. Almada**, **Paola A. Romecin-Duran**, **Eriko Iguchi**, **Aryan Ala’Aldeen**, **Jean-Pierre A. Kocher**, **Gavin R. Oliver**, **Naresh Prodduturi**, **David W. Mead**, **Asif Hossain**, **Norine E. Huneke**, **Colleen M. Tagtow**, **Sikander Ailawadhi**, **Stephen M. Ansell**, **Michaela S. Banck**, **Asher A. Chanan-Khan**, **Ronald S. Go**, **Thorvardur R. Halfdanarson**, **Richard W. Joseph**, **Prashant Kapoor**, **Aaron S. Mansfield**, **Amulya A. Nageswara Rao**, **Grzegorz S. Nowakowski**, **Animesh Pardanani**, **Sameer A. Parikh**, **John C. Cheville**, **Andrew L. Feldman**, **Ramesh K. Ramanathan**, **Steven I. Robinson**, **Heidi D. Finnes**, **Jennifer B. McCormick**, **Robert R. McWilliams**, **Aminah Jatoi**, **Mrinal M. Patnaik**, **Eric D. Wieben**, **Tammy M. McAllister**, **Kandelaria M. Rumilla**, **Sarah E. Kerr**, **Konstantinos N. Lazaridis**, **Gianrico Farrugia**, **Karl J. Clark**, **Eric W. Klee**, and **Martin E. Fernandez-Zapico**, Mayo Clinic, Rochester; **Ernesto Resnik**, Bio-Techne, Minneapolis, MN; **Sikander Ailawadhi**, **Asher A. Chanan-Khan**, and **Richard W. Joseph**, Mayo Clinic, Jacksonville, FL; **Jan B. Egan**, **Alan H. Bryce**, **Estrella M. Carballido**, **Kelly K. Curtis**, **Chelsea D. Gawryletz**, **Thai H. Ho**, **Nathalie Meurice**, **Ramesh K. Ramanathan**, **Raoul Tibes**, **Alvin C. Silva**, **A. Keith Stewart**, and **Mitesh J. Borad**, Mayo Clinic, Phoenix, AZ; and **Eileen J. Kennedy**, University of Georgia, Athens, GA.

## Abstract

**Purpose:**

Genomic testing has increased the quantity of information available to oncologists. Unfortunately, many identified sequence alterations are variants of unknown significance (VUSs), which thus limit the clinician’s ability to use these findings to inform treatment. We applied a combination of in silico prediction and molecular modeling tools and laboratory techniques to rapidly define actionable VUSs.

**Materials and Methods:**

Exome sequencing was conducted on 308 tumors from various origins. Most single nucleotide alterations within gene coding regions were VUSs. These VUSs were filtered to identify a subset of therapeutically targetable genes that were predicted with in silico tools to be altered in function by their variant sequence. A subset of receptor tyrosine kinase VUSs was characterized by laboratory comparison of each VUS versus its wild-type counterpart in terms of expression and signaling activity.

**Results:**

The study identified 4,327 point mutations of which 3,833 were VUSs. Filtering for mutations in genes that were therapeutically targetable and predicted to affect protein function reduced these to 522 VUSs of interest, including a large number of kinases. Ten receptor tyrosine kinase VUSs were selected to explore in the laboratory. Of these, seven were found to be functionally altered. Three VUSs (FGFR2 F276C, FGFR4 R78H, and KDR G539R) showed increased basal or ligand-stimulated ERK phosphorylation compared with their wild-type counterparts, which suggests that they support transformation. Treatment of a patient who carried FGFR2 F276C with an FGFR inhibitor resulted in significant and sustained tumor response with clinical benefit.

**Conclusion:**

The findings demonstrate the feasibility of rapid identification of the biologic relevance of somatic mutations, which thus advances clinicians’ ability to make informed treatment decisions.

## INTRODUCTION

The application of next-generation sequencing techniques to analyze tumor tissue provides opportunities for identifying genes and pathways that drive transformation in individual patients. Precision medicine initiatives use genomic information to facilitate decision making of therapeutic options by defining each patient’s unique tumor mutation landscape. In addition, these data in conjunction with the patient’s clinical data have the potential to increase our knowledge of mechanistic origins, progression, and maintenance of tumors.^1-4^

State-of-the-art techniques can identify mutations on a genome-wide scale, including whole-genome and transcriptome sequencing, as well as more-focused approaches, such as targeted exome sequencing.^5,6^ These procedures have become more affordable and standardized over time. Thus, many institutions and corporations are capable of generating reliable sequencing data for individual patients. Numerous studies have identified defined mutations of known functional significance as biomarkers to predict treatment response and disease prognosis. For example, BRAF V600E is well accepted as a therapeutic target in metastatic melanoma.^7^ However, a current challenge in genomic oncology care is the evaluation of the potential therapeutic significance of large numbers of uncharacterized, nonsynonymous sequence alterations referred to as variants of unknown significance (VUSs) in potentially oncogenic proteins. When novel VUSs are identified, the clinical team must try to draw conclusions about whether the variant is a driver mutation or has no significance for cancer pathogenesis. Although some recurrent mutations within oncogenic proteins have been characterized as having an effect on protein function and thus, the promotion of transformation, novel VUSs identified through clinical genomic testing often are lacking functional information. The definition of the therapeutic value of VUSs is a current unmet need.^8^

We demonstrate how in silico analysis and experimental laboratory studies can rapidly determine the potential therapeutic value of a VUS. By using bioinformatic analysis of exome sequencing results, a large number of potentially deleterious VUSs that are therapeutically targetable were identified with a high frequency of occurrence in kinases. Three-dimensional (3D) modeling of several VUSs located within kinase catalytic domains predicted likely functional significance of these VUSs. Laboratory investigations of a subset of receptor tyrosine kinase (RTK) VUSs defined several functionally altered VUSs. Most significantly, variant F276C in FGFR2 was found to be constitutively activated and sensitive to targeted therapy in vitro. The clinical value of these findings was supported by an observed response to an FGFR inhibitor, BGJ398, in a patient’s tumor that carried FGFR2 F276C. These integrated approaches may provide new avenues to improve personalized treatment of patients with cancer.

## MATERIALS AND METHODS

Detailed materials and methods are available in the Data Supplement.

Patients were referred to the Center for Individualized Medicine (CIM) Oncology Service^9^ between October 2012 and December 2015. Clinical information about these patients was obtained from Mayo Clinic medical records. Informed consent was obtained for each patient who participated in the CIM research protocol approved by the Mayo Clinic institutional review board (IRB 12-007850). The Mayo Clinic IRB approved the in vitro functional studies of somatic mutations identified in tumors of patients enrolled in the CIM Oncology Service (IRB 15-003386). REDCap (Research Electronic Data Capture) hosted at the Mayo Clinic was used to collect and store clinical follow-up data.^10^

## RESULTS

### VUSs Are the Most Common Findings in Tumor Exome Sequencing Analysis

**Fig 1.**
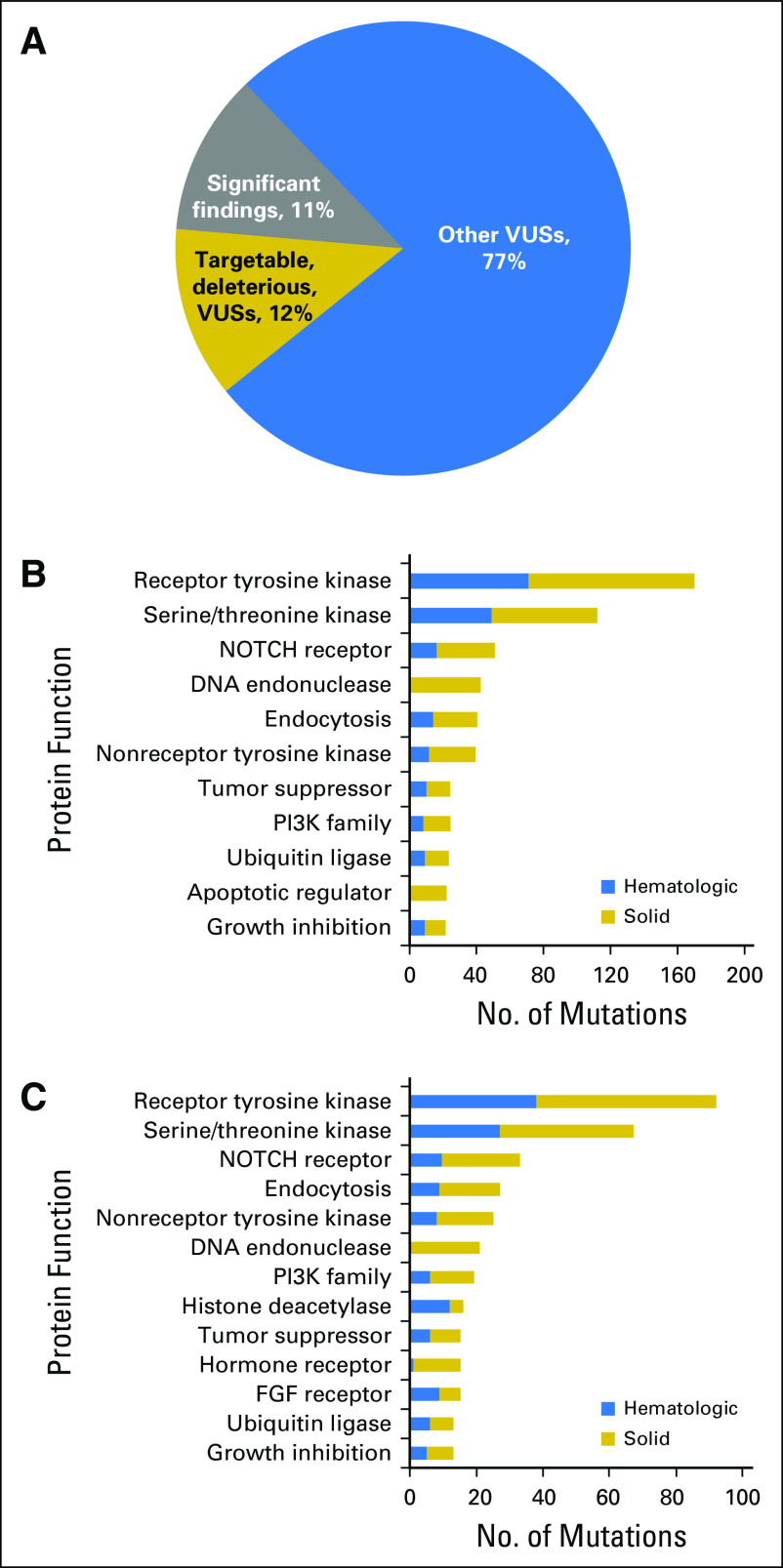
Tumor genomic landscape. (A) Frequency of significant findings and uncharacterized/unreported variants of unknown significance (VUSs) in 4,327 point mutations reported in 308 patient tumors, including solid and hematologic malignancies. (B) Frequency of the most commonly observed protein classes in 905 therapeutically targetable VUSs. (C) Frequency of the most commonly observed protein classes in 522 therapeutically targetable, potentially deleterious VUSs.

Targeted and exome sequencing were performed on heterogeneous solid (57%) and hematologic (43%) malignancies (Data Supplement). More than 4,300 single nucleotide variants were reported from 308 patient tumors (Data Supplement). VUSs, which constitute mutations that are functionally uncharacterized or previously unreported in the COSMIC (Catalogue of Somatic Mutations in Cancer) database,^11^ comprised the majority (89%) of the point mutations observed in this cohort (Fig 1A). Obstacles to clinical use of these data resulted from large numbers of VUSs identified in each patient, uncertainty whether identified VUSs are potential drivers of transformation, and whether encoded proteins were therapeutically targetable.

To begin to address these difficulties, VUSs were filtered into a subgroup of 905 in genes that encode proteins therapeutically targetable by Food and Drug Administration–approved drugs or by investigational agents available through clinical trials. Protein class analysis demonstrated that RTKs and serine/threonine kinases represent the largest therapeutically targetable functional classes, with mutations in both hematologic and solid tumors (Fig 1B).

These 905 therapeutically targetable VUSs were then evaluated in silico, and those determined to be benign by two prediction tools were removed, which left 522 potentially deleterious, therapeutically targetable VUSs in 226 patients (Data Supplement) or 12% of the total variants detected (Fig 1A). The distribution of protein classes represented in this deleterious, therapeutically targetable subgroup (Fig 1C) was similar to that of the therapeutically targetable VUS group (Fig 1B). A large number of the VUSs in this cohort occurred in kinase proteins (Fig 1B). Involvement of kinases in transformation is well established, and Food and Drug Administration–approved drugs/clinical trials that target these pathways are readily available. Of 83 potentially deleterious, therapeutically targetable VUSs for which structural data were available for potential 3D modeling, 41 were in kinases (Data Supplement). From these, six underwent representative 3D modeling with PyMOL software (Schrodinger, New York, NY; Data Supplement), which predicted potentially altered function on the basis of their location within the protein’s kinase domain (Data Supplement).

### In Silico and Functional Characterization of RTK VUSs Identified a Subset of New Targetable Mutations

Ten RTK VUSs from varied solid and hematologic malignancies (Data Supplement) and in regions of functional interest were selected for additional evaluation by in vitro testing. When structural information was available, the RTK VUS of interest underwent 3D modeling to compare wild type (WT) and mutated protein structures. In addition, sequence alignment across species demonstrated that the WT residue that corresponded to each of the 10 VUSs was completely conserved among diverse species, including mouse, rat, bovine, and human proteins. To characterize RTK variants in vitro, FLAG-epitope–tagged mammalian expression constructs were generated for each RTK VUS and its WT counterpart. Cancer cells were transiently transfected to express each VUS and WT RTK, which were compared for cellular localization (by immunofluorescence), expression level (by Western blot for FLAG-tagged RTK), and intracellular signaling (by Western blot of ERK phosphorylation).

**Fig 2.**
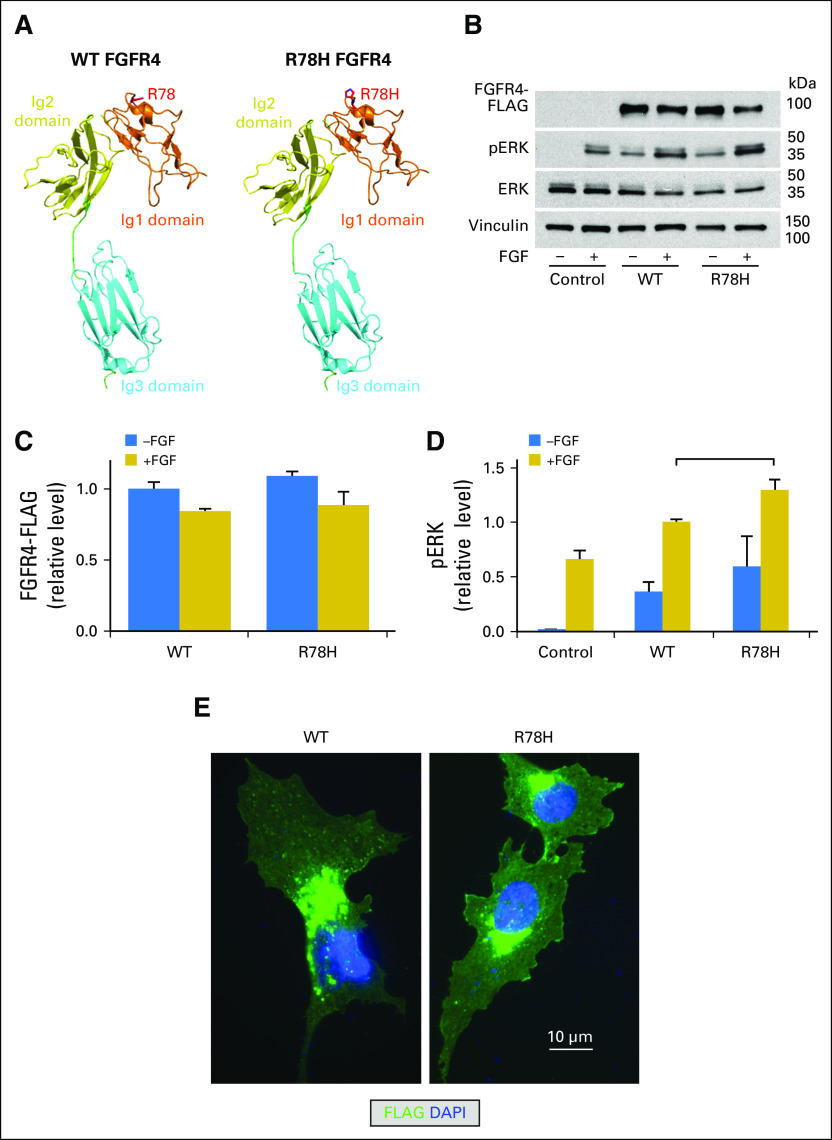
Functional evaluation of FGFR4 variants of unknown significance (VUSs). (A) PyMOL modeling of wild-type (WT; left) and R78H (right) FGFR4. KMCH-1 cells were transfected to express FLAG-tagged WT and R78H FGFR4, or vector only (control). After 1 day, the cells were incubated at 37°C with and without FGF2 in 0.1% bovine serum albumin/DMEM overnight. Right panel: After 1 day, the cells were incubated with and without 20 ng/mL FGF2 in 0.1% bovine serum albumin/DMEM for 16 hours at 37°C. (B) Cell samples were then lysed and subjected to Western blot analysis. Total ERK and vinculin are shown as loading controls. (C) Quantitation of FGFR4 in Western blots (n = 5). Values are mean ± SE normalized to WT (−FGF2) levels. (D) Quantitation of phospho-ERK (pERK) in Western blots (n = 5). Values are mean ± SE normalized to WT (+FGF2) levels. Bracket indicates groups within treatment types (−FGF or +FGF) among FGFR4-transfected samples that were significantly different (*P* < .05) from each other in two-tailed *t* tests. (E) HuCCT-1 cells were transfected with FLAG-tagged WT and R78H FGFR4 for 2 days and then processed for immunofluorescence by using an anti-FLAG antibody. DAPI, 4′,6-diamidino-2-phenylindole; Ig1, immunoglobulin-like domain 1.

FGFR4 variant R78H is located in the extracellular first immunoglobulin-like domain 1 (Ig1). Although Ig2 and Ig3 domains of the receptor are involved in binding FGF, the FGFR4 R78H VUS in the Ig1 is not involved in ligand interaction^2^ (Fig 2A). Biochemical studies of R78H demonstrated no significant differences from WT FGFR4 in overall expression levels when expressed in KMCH-1 cholangiocarcinoma cells (Figs 2B and 2C). Cellular localization of WT and R78H FGFR4 was also similar and exhibited mainly a plasma membrane distribution with intracellular labeling of probable Golgi membranes (Fig 2E). However, R78H FGFR4 exhibited a small, but significantly elevated FGF2-stimulated phospho-ERK (pERK) level compared with WT (Figs 2B and 2D).

**Fig 3.**
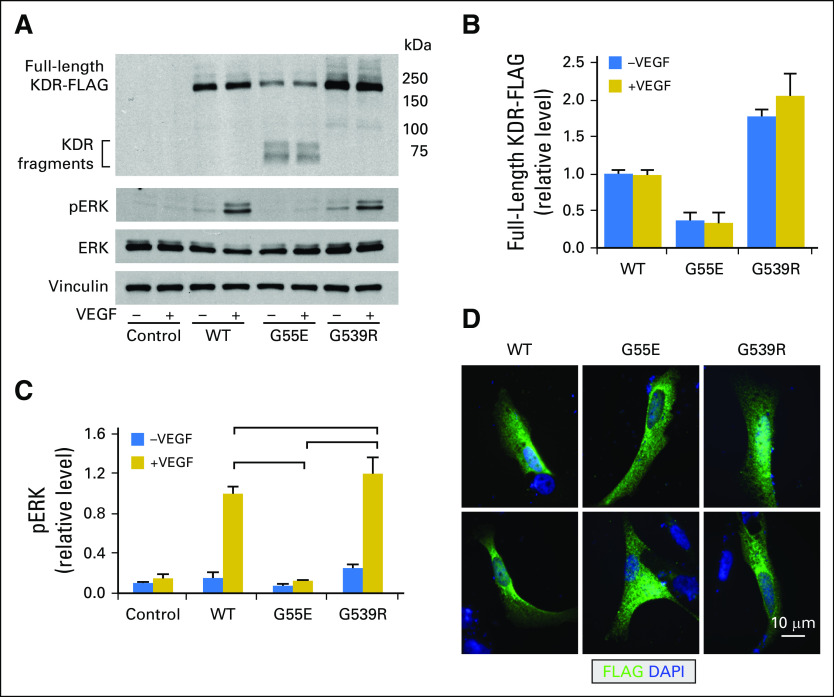
Characterization of KDR variants. (A) KMCH-1 cells were transfected to express FLAG-tagged wild-type (WT), G55E, and G539R KDR, or vector only (control). After 1 day, the cells were serum starved in 0.1% bovine serum albumin/DMEM overnight and then incubated with and without 25 ng/mL vascular endothelial growth factor (VEGF) for 10 min at 37°C. Cell samples were then lysed and subjected to Western blot analysis for FLAG-KDR, phospho-ERK (pERK), total ERK, and vinculin. Equal amounts of total protein were loaded per lane. Note the greater G539R expression and VEGF-stimulated pERK levels than WT. G55E expression of full-length receptor (approximately 200 kDa) was decreased compared with WT, with the appearance of approximately 70- to 80-kDa fragments, which suggested decreased stability/increased degradation of the G55E form. pERK was barely detectable in G55E samples and not increased by VEGF treatment. (B) Quantitation of full-length KDR levels from Western blots (n = 3). Values are mean ± SE and were normalized to WT (−VEGF) levels. All samples were significantly different (*P* < .05) from others in the same treatment group (−VEGF or +VEGF) in two-tailed *t* tests. (C) Quantitation of pERK levels from Western blots (n = 3). Values are mean ± SE and were normalized to WT (+VEGF) levels. Brackets indicate groups within treatment types (−VEGF or +VEGF) among KDR-expressing samples that were significantly different (*P* < .05) from one another in two-tailed *t* tests. (D) HuCCT-1 cells were transfected with FLAG-tagged WT and variants of unknown significance KDR for 2 days and then processed for immunofluorescence with an anti-FLAG antibody. Two examples of each KDR protein are shown. DAPI, 4′,6-diamidino-2-phenylindole.

Two KDR VUSs, G55E and G539R, are located in the extracellular domain in proximity to amino acids that form disulfide bonds (C53 and C530); thus, these amino acid substitutions may potentially affect neighboring disulfide bonds. Expression of WT and VUS KDR proteins in KMCH-1 cells demonstrated that G539R KDR was more highly expressed than WT (Figs 3A and 3B). In contrast, G55E exhibited decreased expression of full-length (approximately 200 kDa) KDR compared with the WT form, with the appearance of a novel approximately 70- to 80-kDa polypeptide doublet in this variant as detected with FLAG antibody (Fig 3A), which suggests that G55E KDR is less stable than the WT protein. Cells that expressed G539R KDR exhibited increased pERK levels upon treatment with vascular endothelial growth factor compared with the WT form, whereas almost no pERK was detected in cells that expressed G55E KDR (Figs 3A and 3C). KDR variants and WT exhibited a similar punctate distribution by immunofluorescence (Fig 3D).

VUSs G251E, V484M, and T643M of PDGFRA and variants V258L and V316M of PDGFRB were investigated. All variants fall in the extracellular domain of their respective receptor except T643M, which occurs within the PDGFRB kinase domain. V258L was predicted to be functionally benign but was of interest for in vitro study because of its Ig3 location. PDGFR VUSs were generally similar to their WT counterparts in terms of expression, distribution of polypeptide species on Western blots, PDGF-stimulated pERK levels, and cellular localization (Data Supplement), except that PDGFRA V484M was significantly lower in expression and PDGF-stimulated pERK levels than WT (Data Supplement) and PDGFRB V316M was significantly lower in expression than WT (Data Supplement).

### F276C Mutant Is a New, Constitutively Active Form of FGFR2

**Fig 4.**
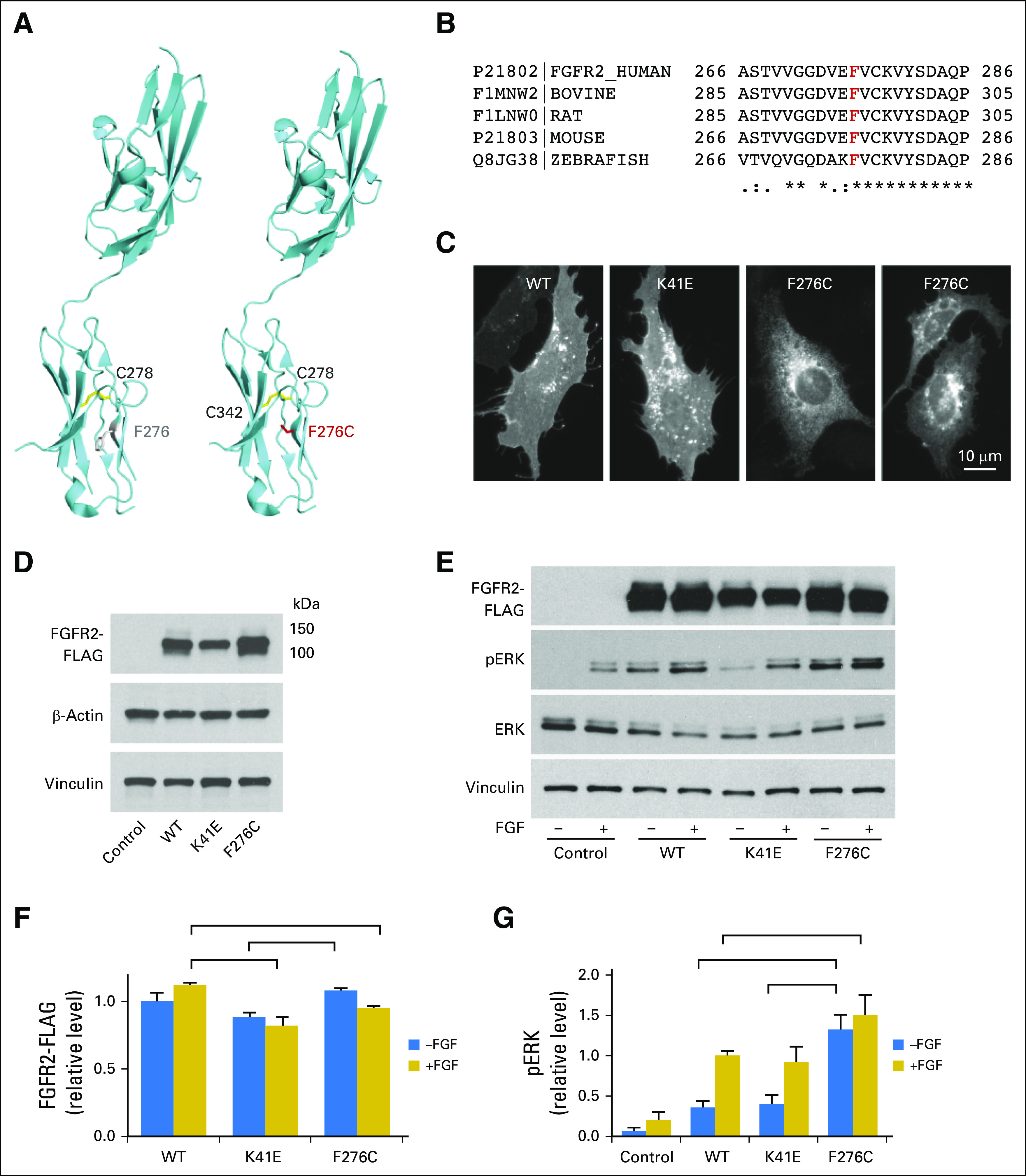
Altered expression and signaling of FGFR2 variants. (A) PyMOL modeling of wild-type (WT; left) and F276C (right) FGFR2 proteins. The extracellular receptor of FGFR2 contains an intrinsic disulfide bond between C278 and C342 in the immunoglobulin-like domain 3 (Ig3; shown in gold). Residue F276 is highlighted in gray and is proximal to the disulfide bridge. The FGFR2 F276C variant (highlighted in red) may lead to the introduction of aberrant disulfide bonds that could alter the activation state of the protein. (B) Sequence alignment shows that residue F276 is highly conserved among the FGFR2 family from zebrafish to humans (sequence alignment performed by using Clustal Omega [EMBL-EBI, Wellcome Genome Campus, UK]; UniProtKB entry numbers are shown). (C) HuCCT-1 cholangiocarcinoma cells transfected with the WT, K41E, and F276C FGFR2 forms for 2 days were fixed, permeabilized, and processed for immunofluorescence by using the FLAG antibody. Images show typical cellular localization of the WT, K41E, and F276C FGFR2 proteins. (D) KMCH-1 cells were transfected with FGFR2 forms by using equal amounts of DNA. After 1 day, cells were switched to serum-free medium and incubated for an additional 16 hours before lysis. Lysates were analyzed for expression of FGFR2 by using an antibody against the FLAG-tag. Housekeeping proteins (β-actin and vinculin) were also detected. Equal total protein (5 μg) was loaded per lane. (E) Functional testing of FGFR2 signaling. KMCH-1 cells were transfected with FGFR2 forms by using a 3.5/5 ratio of F276C/WT and K41E DNA to adjust expression of the F276C protein to similar levels as the WT form. After 1 day, cells were switched to serum-free medium with or without 20 ng/mL FGF2 and incubated an additional 16 hours at 37°C before lysis. Lysates were analyzed by Western blot for expression of FGFR2-FLAG, phospho-ERK (pERK), total ERK, and vinculin. (F) Quantitation of FGFR2 levels in Western blots as in (E). Values are mean ± SE from three experiments normalized to WT (−FGF2) levels. (G) Quantitation of pERK levels in Western blots as in (E). Values are mean ± SE from three experiments normalized to WT (+FGF2) levels. Brackets indicate groups within treatment types (−FGF or +FGF) among FGFR2-expressing samples that were significantly different (*P* < .05) from one another in two-tailed *t* tests. In (G), pERK levels from all FGFR2-transfected groups were significantly different from each control group.

Although predicted in silico to be benign, FGFR2 K41E, identified in an acute myeloid leukemia, was selected for additional study because of its location in the extracellular Ig1 of FGFR2 and its unknown effect on ligand binding in nearby Ig2 and Ig3 (Data Supplement). The FGFR2 F276C mutation identified here from a cholangiocarcinoma was also in a single cholangiocarcinoma in the COSMIC database but has not been characterized.^11,12^ F276C is located in an extracellular, Ig-like C2-type 3 domain^13^ where ligand binding occurs.^14^ A different amino acid substitution at the same residue, F276V, has been reported in Crouzon syndrome.^15^ Modeling of WT and F276C FGFR2 showed that the extracellular receptor of FGFR2 contains an intrinsic disulfide bond between C278 and C342 in Ig3 (Fig 4A, shown in gold). Residue F276 is proximal to the disulfide bridge, which suggests that F276C disrupts normal disulfide linkages. Alignment of residue F276 is highly conserved from zebrafish to humans, which suggests that its alteration disrupts normal protein function (Fig 4B). These data suggest that the F276C variant is functionally altered relative to the WT form.

When FGFR2 proteins were expressed in KMCH-1 cells, immunofluorescence microscopy showed that the WT and K41E FGFR2 proteins were localized mainly to the cell surface and occurred on intracellular structures, likely endosomes and the Golgi apparatus (Fig 4C). In contrast, the F276C variant exhibited an endoplasmic reticulum–like appearance and bright Golgi-like intracellular structures in most cells, with only a minority of cells showing an obvious plasma membrane distribution (Fig 4C). Each protein was expressed as an approximately 130-kDa polypeptide as detected by Western blot (Fig 4D). However, the F276C variant was expressed at a higher level and the K41E variant at a lower level compared with WT (Fig 4D), although cells were transfected with equal amounts of DNA for the different FGFR2 constructs.

Functional characteristics of FGFR2 proteins were assessed by studying pERK signaling. Because the F276C FGFR2 variant is expressed at higher levels than WT when equal amounts of DNA were used, a lower ratio of F276C construct DNA was used for transfection in the following experiments so that resulting levels of WT and F276C FGFR2 proteins were comparable (Figs 4E and 4F): KMCH-1 cells transfected with FGFR2 constructs were treated for 16 hours in serum-free media with FGF2, lysed, and analyzed by Western blot. In the absence of FGF2, control transfected cells exhibited negligible levels of pERK, and pERK was increased by FGF2 treatment (Figs 4E and 4G). The expression of WT or K41E FGFR2 in the absence of FGF2 increased pERK levels beyond control levels, and treatment with FGF2 led to approximately fivefold increases in pERK compared with control for both WT and K41E. However, expression of F276C FGFR2 significantly increased the pERK level in the absence of FGF2 compared with WT FGFR2, with little increase upon treatment with FGF2 (Figs 4E and 4G). Similarly high constitutive activity of F276C versus WT was observed in PANC1 cells and KMBC cholangiocarcinoma cells. In summary, F276C FGFR2 has high expression, altered cellular distribution, and increased constitutive activity compared with WT.

**Fig 5.**
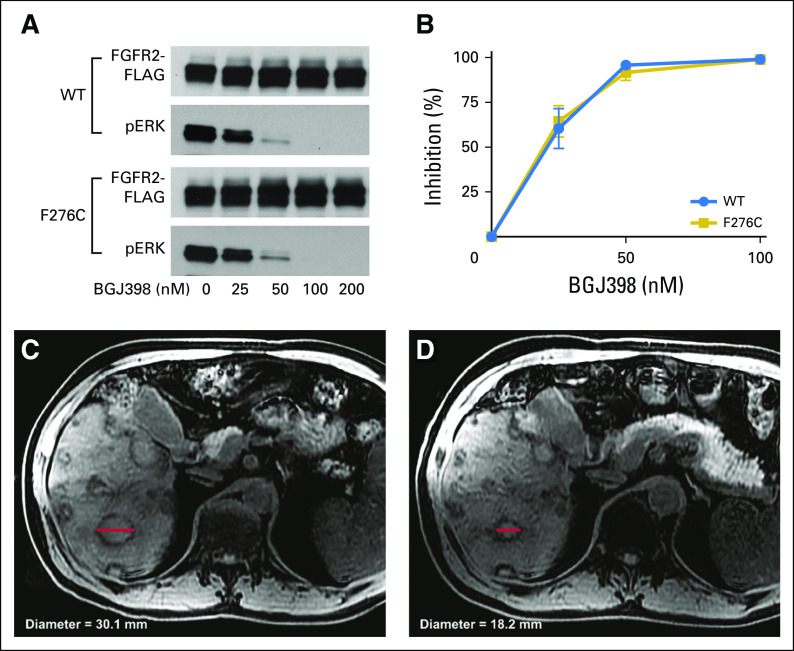
Response of F276C FGFR2 to BGJ398 treatment. (A) KMCH-1 cells were transfected with F276C and wild type (WT) by using a 3.5/5 DNA ratio, respectively, to normalize expression levels. After 1 day, cells were incubated with serum-free medium with 20 ng FGF2 for 16 hours. Cells were then treated with BGJ398 (0 to 200 nM) or vehicle (dimethyl sulfoxide) at 37°C for 3 hours. Cell lysates were then analyzed by Western blot for FGFR2-FLAG and phospho-ERK (pERK). (B) Quantitation of dose response to BGJ398 for experiments as shown in (A); n = 3 for each BGJ398 concentration. Values are mean ± SE and expressed as percent inhibition of pERK signal compared with cells with no BGJ398. Response of FGFR2 F276C–containing tumor to BGJ398 in (C) September 2015 (pretreatment, with magnetic resonance imaging showing a 30.1-mm tumor diameter [red line]) and (D) October 2015 (postinitiation of treatment, with pan-FGFR inhibitor BGJ398 magnetic resonance imaging showing tumor shrinkage to an 18.2-mm diameter [red line]).

Finally, the sensitivity of WT and F276C FGFR2 activities to treatment with the FGFR inhibitor BGJ398 were compared. KMCH-1 cells were transfected with WT or F276C constructs and were incubated for 16 hours the next day with FGF2 in serum-free media. Cells were then treated for 3 hours with a range of concentrations of BGJ398 or dimethyl sulfoxide control, after which cells were lysed and analyzed for pERK levels. At concentrations between 0 and 100 nM, ERK phosphorylation was similarly partially inhibited by BGJ398 in cells that expressed WT or F276C, and both FGFR2 forms were completely inhibited by BGJ398 at 200 nM (Figs 5A and 5B). These studies demonstrate that the F276C FGFR2 variant has comparable sensitivity to BGJ398 relative to WT.

### Clinical Response to FGFR Inhibitor in a Patient With Cholangiocarcinoma Who Carried Somatic F276C VUS

The FGFR2 F276C VUS was identified as a result of targeted sequencing of a tumor from a 57-year-old male with advanced, multifocal, intrahepatic cholangiocarcinoma. He was originally treated in a clinical trial with gemcitabine, cisplatin, and silmitasertib, which resulted in a partial response. He remained on this treatment of 10 months, at which time his disease progressed and he was switched to capecitabine and oxaliplatin. Two months later, the disease became refractory to that regimen. Consequently, a 600-gene next-generation sequencing panel was obtained for the patient’s tumor (Caris Molecular Intelligence, Phoenix, AZ). The FGFR2 F276C VUS was reported and confirmed to be a somatic event (data not shown). On the basis of this finding, the patient started on BGJ398 (ClinicalTrials.gov identifier: NCT02150967), achieved a partial response to therapy after 2 months (Figs 5C and 5D), and maintained the response for an additional 4 months, at which time new lesions developed that led to discontinuation of BGJ398. As a result of our in vitro studies of F276C, a mechanism of action has now been correlated with this observed clinical response of the tumor to BGJ398.

## DISCUSSION

The volume of new data from individual and group sequencing efforts (eg, The Cancer Genome Atlas) has rapidly expanded the understanding of the incidence and frequency of mutations in disparate cancers. The translation of these data to affect treatment choices and patient care remains a significant challenge. Few mutations have extensive preclinical and clinical evidence that support the effectiveness of targeted therapies, and genomic testing often reveals that tumors have numerous VUSs, including variant sequences for which no functional data are available. Mutations that have not been functionally characterized present a significant and growing challenge to the treating physician. In the absence of clinical trial or even preclinical data, the question becomes how to quickly assess the potentially deleterious effect of VUSs that occur in therapeutically targetable genes. In vitro functional studies can be conducted but often require weeks for return of results compared with the hours or days preferred in the clinical setting. With multiple uncharacterized/unreported mutations returned for each patient, a method of prioritizing which variants to study in depth is necessary.

We addressed this problem by developing an approach that uses an in silico filtering process to prioritize mutations of the highest biologic and clinical interest. From 3,833 VUSs, a subset of 522 was generated that encoded potentially targetable proteins likely to be functionally altered, including numerous kinases. From this list, 10 RTK VUSs were selected for further characterization. Of these 10 variants, seven were found to be altered in expression or activity relative to the WT protein, and three of these (FGFR2 F276C, FGFR4 R78H, and KDR G539R) demonstrated greater activity than their WT counterparts (Table 1), which suggests that these mutations play a role in promoting oncogenesis. In contrast, four VUSs (FGFR4 K41E, KDR G55E, PDGFRA V484M, and PDGFRB V316M) exhibited reduced expression compared with their WT counterparts, and thus were unlikely to be involved in oncogenesis in the tumors where they occurred. These findings not only support the strength of our in silico analysis in predicting whether VUSs are functionally altered but also point to the inability to distinguish among activating, deactivating, and destabilizing mutations.

**Table 1.**
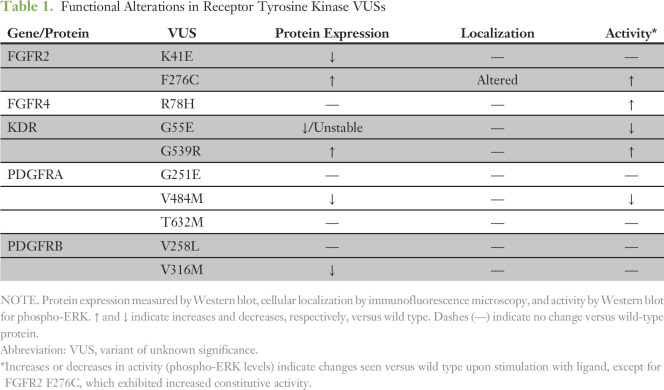
Functional Alterations in Receptor Tyrosine Kinase VUSs

Optimization of appropriate cellular models is important in methods development for functional evaluation of VUSs. This work was largely conducted by using KMCH-1 cholangiocarcinoma cells because of these cells’ high transfectability and extremely low basal pERK levels. Readouts for functional alterations were based on expression levels of the VUS and effects on pERK signaling, which enabled rapid assessment. For more understanding of the functional significance of VUSs, these studies should be followed by experiments that use cell types that match the VUS tumor of origin and evaluate end points such as cell growth and viability.

FGFR2 F276C was identified as a VUS of potential interest because of the presence of several factors, including prediction of a deleterious effect by two algorithms, 3D modeling that suggests an increase in activity on the basis of its location in the ligand-binding Ig3, and proximity to a key disulfide bond. Germline mutations of residues to and from cysteine in this region of FGFR2 (eg, Y328C, C278F) have been reported to allow the formation of aberrant disulfide bonds and to induce constitutive receptor dimerization and activation, which lead to a variety of skeletal and craniosynostosis disorders (eg, Crouzon and Pfeiffer syndromes).^16-18^ Our molecular modeling combined with the demonstration that F276C FGFR2 is more highly expressed and constitutively active than the WT receptor suggests that this mutation alters disulfide bonds, which alters receptor dimerization and activity similarly to the FGFR2 mutations seen in craniosynostosis syndromes. By using in vitro studies, we show that the F276C FGFR2 variant is sensitive to BGJ398, a pan-FGFR inhibitor, which was also reflected clinically in the response of a patient’s tumor when treated with BGJ398 as part of a clinical trial (Fig 5). These data suggest that F276C is a therapeutically targetable mutation. However, additional studies, such as the testing of BGJ398 effectiveness in impeding growth of organoids or xenografts that express WT versus F276C, are needed to confirm that FGFR2 F276C is actionable.

The ability to identify potentially actionable VUSs from numerous VUSs for each patient tumor would simplify therapeutic choices. In silico analysis requires only hours to conduct and as demonstrated here, can yield a subset of VUSs that encode therapeutically targetable proteins likely to be altered in function by their variant sequence. In vitro functional studies are more time consuming and may not fit practically within a patient’s progression timeline, but they may yield more-definitive findings. Ideally, attempts should be made to develop publically available databases of anonymized patient VUS profiles and responses to targeted therapies as well as VUS databases with information on in vitro characterization of functional alterations and responses to targeted pharmaceuticals.^8,19^ Oncologists and their patients with cancers that are a challenge to treat could greatly benefit from these resources.
